# Smoking and antidepressants pharmacokinetics: a systematic review

**DOI:** 10.1186/s12991-017-0140-8

**Published:** 2017-03-06

**Authors:** Pedro Oliveira, Joana Ribeiro, Helena Donato, Nuno Madeira

**Affiliations:** 1 Psychiatry Department, Coimbra Hospital University Centre, Praceta Mota Pinto, 3000-075 Coimbra, Portugal; 2 Documentation Department, Coimbra Hospital University Centre, Coimbra, Portugal

**Keywords:** Antidepressant agents, Pharmacokinetics, Depressive disorder, Smoking

## Abstract

**Background:**

Despite an increasingly recognized relationship between depression and smoking, little is known about how smoking influences antidepressant response and treatment outcomes. The aim of this study was to systematically review the evidence of the impact of smoking on new-generation antidepressants with an emphasis on the pharmacokinetic perspective.

**Methods:**

We present a systematic review of clinical trials comparing the serum levels of new-generation antidepressants in smokers and nonsmokers. Data were obtained from MEDLINE/PubMed, Embase, and other sources. Risk of bias was assessed for selection, performance, detection, attrition, and reporting of individual studies.

**Results:**

Twenty-one studies met inclusion criteria; seven involved fluvoxamine, two evaluated fluoxetine, sertraline, venlafaxine, duloxetine or mirtazapine, and escitalopram, citalopram, trazodone and bupropion were the subject of a single study. No trials were found involving other common antidepressants such as paroxetine or agomelatine. Serum levels of fluvoxamine, duloxetine, mirtazapine and trazodone were significantly higher in nonsmokers compared with smokers.

**Conclusions:**

There is evidence showing a reduction in the concentration of serum levels of fluvoxamine, duloxetine, mirtazapine and trazodone in smoking patients as compared to nonsmokers. The evidence regarding other commonly used antidepressants is scarce. Nonetheless, smoking status should be considered when choosing an antidepressant treatment, given the risk of pharmacokinetic interactions.

## Background

One-quarter of the general population, 40–50% of people with depression and 70–80% of those with schizophrenia smoke [1, 2]. Major depressive disorder (MDD) is a common psychiatric disease and a major public health problem. It has been projected to become the leading cause of disability and the second leading contributor to the global burden of disease and overall mortality by the year 2020; according to the recent epidemiologic data, about 10% of world population suffers with depression [3]. The advent of antidepressants (AD), starting with imipramine in 1958, has revolutionized the treatment of depression; yet this first generation of AD had an unfavorable adverse effect profile that has improved significantly with the advent in the late 1980s of selective serotonin reuptake inhibitors (SSRI) and, some years later, of serotonin and noradrenaline reuptake inhibitors (SNRI) and others like mirtazapine, trazodone, agomelatine and bupropion [4]. Given their favorable adverse effects profile, new-generation antidepressants are the first-line treatment of MDD. The mechanism of action and efficacy is similar between drugs of the same pharmacological class. However, there are considerable differences among interindividual responses to a given drug. Many theories tried to explain this variation but the most accepted is that clinical response is related to serum levels of the antidepressants. For many SSRI and other new-generation antidepressant drugs, a relationship between plasma concentrations and clinical effects is not reported [5]. However, serum levels below therapeutic range could compromise the clinical response [5]. The serum levels of antidepressants are essentially dependent on two variables: the intake dose of drug and the rate of elimination. The rate of elimination of antidepressants is almost exclusively dependent on their hepatic metabolization. The hepatic metabolism of AD is made by cytochrome P450 (CYP) which is a group of many enzymes that exists in different amounts in human liver. Different antidepressants are metabolized by different subtypes of CYP and the amount of each CYP varies from person to person [6]. Thus, it is now accepted that the difference found in the response to an antidepressant could be correlated with serum concentrations. With the exception of tricyclic antidepressants, the correlation between plasmatic levels and the clinical outcome is still not consensual [7]. However, AGNP Consensus Guidelines from 2011 attribute a level 2 of recommendation for therapeutic drug monitoring for SSRI (except paroxetine) and SNRI [7]. Despite the availability of techniques capable of quantifying the activity of CYP isozymes such as 1A2, 2B6, 2C9 and 3A4 [8], they are not commonly used in clinical practice. So, given the absence of clinical indicators as relating to the higher or lower individual activity of each CYP, often the choice is made based on clinical experience with the particular drug and previous response of the individual to antidepressants in the past. Such imprecision can lead to the inappropriate use of drugs, not only wasting time until an adequate remission of depression but also causing iatrogenic harm, jeopardizing patient trust in antidepressant treatments.

There are 4000 chemical compounds found in cigarette smoke and 43 have been identified to be carcinogenic. Cigarette smoke constituents have been shown to stimulate or induce hepatic CYP isozymes, which play a central role in drug metabolism. Polycyclic aromatic hydrocarbons (PAH) from cigarette smoke are responsible for the induction of CYP isozymes. PAHs have been shown to induce CYP1A1, CYP1A2 and CYP2E1 [9].

Given the possibility that some antidepressants are metabolized by CYP induced or inhibited by substances in tobacco, their identification can be a guide for drug initial choice in smoking patients, allowing a more accurate antidepressant selection and consequently improving the pharmacologic treatment of depression in smokers.

Cigarette smoking can affect the clinical management of patients with psychiatric disorders because of the pharmacokinetic and pharmacodynamical changes; it can cause to various psychotropic drugs. This article reviews the impact of smoking on new-generation antidepressants with an emphasis on the pharmacokinetic perspective. It also seeks to provide critical information on whether such variations should influence antidepressant choice in this population.

## Methods

This review was performed according to the PRISMA guidelines [10], thus providing a comprehensive framework which objectively assesses indicators of quality and risk of biases of included studies.

All original studies investigating the difference between the levels of any new-generation antidepressive agents (SSRI, SNRI, trazodone, mirtazapine, bupropion or agomelatine) and the smoking status were eligible for this systematic review. Further criteria adopted were: (1) publication date between January 1970 and June 2016, (2) empirical study, (3) written in English or Portuguese language, (4) published in a scholarly peer-reviewed journal, (5) studies that determined serum levels within a steady state, and (6) comparison of serum levels between 2 groups—smokers and nonsmokers. Additionally, studies were excluded from review if they were: (1) single-case report, (2) review articles, (3) repeated study population, (4) comparisons involving combinations of drugs, (5) animal studies, and (6) trials involving only metabolites.

**Fig. 1:**
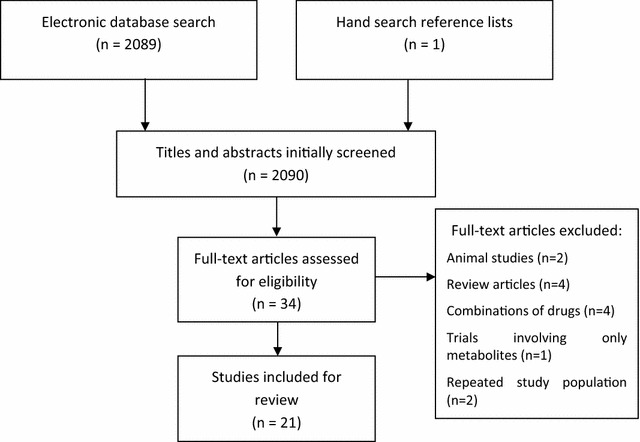
PRISMA flow diagram of the study selection process

Studies were identified by searching relevant papers via PubMed/MEDLINE (http://www.ncbi.nlm.nih.gov/pubmed), Cochrane Library and EMBASE using the following keywords: (“antidepressive agents”) AND (smok*). Finally, reference lists of retrieved studies were hand searched to identify any additional relevant studies. Keywords and combination of keywords were used to search the electronic databases and were organized following the population intervention comparison outcome (PICO) model (Fig. 1). In this model, the search strategy can be organized based on the topics: population (P), intervention (I), control group (C), and outcome (O) and several searches in the aforementioned databases.

After performing the initial literature searches, each study title and abstract was screened for eligibility by the first author. Full text of all potentially relevant studies were subsequently retrieved and further examined for eligibility. The PRISMA flow diagram (Fig. 1) provides more detailed information regarding the selection process of studies. Information from the included studies was then analyzed and recorded in an electronic spreadsheet designed by the first author. Different types of data were extracted from each study including: (a) country in which the data were collected and participants’ characteristics, (b) number of subjects, (c) number of smokers, (d) age average, (e) percentage of males, (f) main results (g) intervention protocol, (h) risk of bias in individual studies, and (i) limitations among others. The Cochrane Collaboration’s tool for assessing risk of bias was adopted to evaluate the risk of bias in individual studies [11]. The following risk of biases was analyzed: (1) selection bias, (2) performance bias, (3) detection bias, (4) attrition bias, and (5) reporting bias.

## Results

Twenty-one articles were included in this review: seven are about fluvoxamine (FLV) [18–24], two about fluoxetine (FLX) [12, 13], sertraline [14, 15], venlafaxine (VEN) [25, 26], duloxetine [27, 28] and mirtazapine [30, 31], one about escitalopram [16], citalopram [17], trazodone [29] and bupropion [32]. No studies were found about paroxetine, milnacipran or agomelatine.

Eight studies were from Sweden, six were from Japan, four from Germany, two were from the United States of America (USA), and one from France and Switzerland. The studies reviewed included 2375 participants of which 733 were smokers. In terms of gender distribution, the vast majority of the studies reviewed recruited more female participants (64.31%) than male participants (35.69%). The average age of the subjects included in the studies is 45.53 years. A summary of results is given in Table 1 and the risk of bias in individual studies based on Cochrane Collaboration’s tool for assessing risk of bias is given in Table 2. As shown in Table 2, selection and reporting bias are the most frequent, with seven of twenty-one studies assessed with high risk for selection bias and four with high risk of reporting bias. No detection and attrition bias were found, although the risk of bias was not always clear.

**Table 1:** Impact of smoking on main antidepressants (SSRI and SNRI) pharmacokinetics

Study	Drug	Country	N total/N smokers	Age	Men (%)	Main findings	Study limitations
Lundmarck et al. [12]	Fluoxetine	Sweden	291/138	43	39	No significant correlation between serum levels of FLX and smoking habits was found	Possibility of interactions with other medications
Koelch et al. [13]	Fluoxetine	Germany	65/13	14.6	37.0	Serum concentrations of the active moiety and NORFLX were significantly correlated with smoking status	Possibility of interactions with other medications
						Serum levels of the active moiety of FLX were 44% lower in smokers than in nonsmokers	Due to the ethnic polymorphism of CYP2D6 [6], the current results are not transferable to races other than Caucasians
						No significant correlation between serum levels of FLX and smoking habits was found	Population younger than 19
Lundmarck et al. [14]	Sertraline	Sweden	319/89	54.4	31	Smokers had significantly lower concentration-to-dose (C/D) mean ratios of serum sertraline and its main metabolite desmethylsertraline than nonsmokers	Possibility of interactions with other medications
Taurines et al. [15]	Sertraline	Germany	85/5	14.8	45.1	No significant correlation between serum levels of Sertraline and smoking habits was found	Possibility of interactions with other medications
							No standardization of timing of blood withdrawal and length of treatment as well as patient compliance
							Low number of subjects taking high doses of sertraline
							Population younger than 19
Reis et al. [16]	Escitalopram	Sweden	130/31	51	32	No significant correlation between serum levels of Escitalopram and smoking habits was found	76% of the patients took one or more drugs in addition to escitalopram
Reis et al. [17]	Citalopram	Sweden	19/10	<21	18.8	No significant correlation between serum levels of Citalopram and smoking habits was found	Possibility of interactions with other medications
							Population younger than 21
Spigset et al. [18]	Fluvoxamine	Sweden	24/12	36.7	58.3	Cmax, and AUC were significantly lower in the smokers than in the nonsmokers	Only 50 mg/day of FLV was tested
						There were no group differences in elimination half-life	Small sample size
Carrillo et al. [19]	Fluvoxamine	Sweden	14/6	33.9	50	Among extensive metabolizers (CYP1A2 e CYP 2D6) there was no difference in FLV kinetics between smokers and nonsmokers	Only 50 mg/day of FLV was tested
							Small sample size
							Limited to extensive metabolizers
Yoshimura et al. [20]	Fluvoxamine	Japan	30/11	52	36.7	Serum levels of FLV were significantly higher in nonsmokers than smokers	Small sample size
							Some subjects also took benzodiazepines
Gerstenberg et al. [21]	Fluvoxamine	Japan	49/15	49.9	69.0	No significant difference between nonsmokers and smokers in the Css of FLV and FLA and FLA/FLV ratio was found	Thirty-nine patients also took benzodiazepines
							Small sample size
Sugahara et al. [22]	Fluvoxamine	Japan	49/13	w.i.	w.i.	The mean C/D ratio of FVX in smokers was reduced by more than 30% in comparison with that in nonsmokers	No information about age or sex of patients
Katoh et al. [23]	Fluvoxamine	Japan	32/6	39.0	46.9	The steady-state plasma C/D ratio of FLV in patients who smoked was significantly lower than that in nonsmoker patients	Possibility of interactions with other medications
							Small sample size
Suzuki et al. [24]	Fluvoxamine	Japan	87/22	36.6	65.5	Heavy smokers had significantly lower FLV concentration than nonsmokers in the FLV 50 mg/d dose group	Possibility of interactions with other medications
						At 150 mg/day and 200 dose groups, no significant differences in FLV concentration were observed between nonsmokers and heavy smokers	Small sample size
Reis et al. [25]	Venlafaxine	Sweden	141/58	61.3	33	The steady-state plasma C/D ratio of ODV and DDV in patients who smoked was significantly lower than that in nonsmoker patients	Possibility of interactions with other medications
						No differences in C/D VEN values or in any of the metabolite/VEN ratios were found	
Unterecker et al. [26]	Venlafaxine	Germany	227/87	49.1	36.4	In smokers, mean serum levels of ODV were 21% lower than in nonsmokers	Possibility of interactions with other medications
						No differences in C/D VEN values between two groups	
Fric et al. [27]	Duloxetine	Germany	23/8	47.3	75	Smokers show significantly lower duloxetine serum level than nonsmokers	Possibility of interactions with other medications
							Small sample size
Lobo et al. [28]	Duloxetine	USA	594/123	48.8	26	Serum levels of duloxetine were significantly lower in smokers than in nonsmokers	Possibility of interactions with other medications
						Nonsmokers have a 43% higher Css than smokers	
Ishida et al. [29]	Trazodone	Japan	43/16	43	44.2	Smokers show significantly lower duloxetine serum level than nonsmokers	Small sample size
						No differences in mCPP concentrations between two groups	Some subjects also took benzodiazepines
Lind et al. [30]	Mirtazapine	Sweden	56/36	50	34	Smokers show significantly lower S-mirtazapine and R-N-desmethylmirtazapine serum levels than nonsmokers	No information about the number of cigarettes consumed daily
Sirot et al. [31]	Mirtazapine	France/Switzerland	45/17	51	18.9	Smokers show significantly lower mirtazapine, S-mirtazapine and R-N-desmethylmirtazapine serum levels than nonsmokers	Possibility of interactions with other medications
Hsyu et al. [32]	Bupropion	USA	34/17	26.2	52.9	No significant correlation between serum levels of bupropion and smoking habits was found	Almost every subjects are caucasian
							Small sample size
							Only evaluates 150 mg daily

**Table 2:** Assessment of risk of bias in individual studies

Study	Selection bias	Performance bias	Detection bias	Attrition bias	Reporting bias
Lundmarck et al. [12]	−	−	−	?	−
Koelch et al. [13]	?	?	−	−	+
Lundmarck et al. [14]	−	−	−	?	−
Taurines et al. [15]	?	?	−	−	+
Reis et al. [16]	?	?	−	?	+
Reis et al. [17]	+	?	−	?	?
Spigset et al. [18]	−	−	−	−	−
Carrillo et al. [19]	−	−	−	−	?
Yoshimura et al. [20]	−	+	−	−	?
Gerstenberg et al. [21]	−	−	−	?	?
Sugahara et al. [22]	?	?	−	−	?
Katoh et al. [23]	+	−	−	−	?
Suzuki et al. [24]	+	−	−	−	−
Reis et al. [25]	+	?	−	?	?
Unterecker et al. [26]	−	−	−	?	−
Fric et al. [27]	+	−	−	?	−
Lobo et al. [28]	−	−	−	−	−
Ishida et al. [29]	?	−	−	−	−
Lind et al. [30]	−	−	−	−	−
Sirot et al. [31]	+	−	?	−	−
Hsyu et al. [32]	+	−	−	−	+

+ high risk of bias, – low risk of bias, ? unclear risk of bias

## Discussion

As mentioned above, tobacco interferes with drug metabolism essentially by the action of PAH that have effects through the induction of CYP 1A1, CYP1A2 and CYP2E1 [9].

All the antidepressants evaluated are metabolized in the liver by different types of cytochromes. With respect to SSRIs, citalopram is metabolized by CYP 2C19 and 3A4 [33], fluoxetine by 2D6, 3A4 and 2C9 [33], fluvoxamine by 1A2 and 2D6 [33], escitalopram by 2C19, 2D6 and 3A4 [16], and sertraline by 2D6, 3A4, 2C9 and 2C19 [33]. Regarding SNRI, venlafaxine is metabolized by CYP 2D6, 3A4 and 2C9 [33], and duloxetine by 2D6 and 1A2 [27]. Trazodone is metabolized by CYP 2D6 [29], mirtazapine by 1A2, 2D6 and 3A4 [31], and bupropion by 2B6 [32].

SSRI, except fluvoxamine, have little research on the effects of tobacco consumption in their serum levels. The trials performed with sertraline, escitalopram and citalopram showed no influence of smoking on their pharmacokinetics. However, the study on citalopram was based on a population in a restricted age group (all subjects were younger than 21 years) and studies regarding sertraline and escitalopram were not randomized and had many important limitations like the possibility of interactions with other drugs. The fluoxetine concentrations did not differ between the two study groups but the levels of its active metabolite, norfluoxetine, were higher in the group of smokers. Since norfluoxetine is an active metabolite, such association may impact the response of patients to take fluoxetine as well as implications with increased half-life of this molecule, which is already long, for example, by drug bioaccumulation and the ability to induce serotonin syndromes. Fluvoxamine has the greater evidence of decreased serum levels when associated with tobacco consumption. Although not proved, most studies suggest that there may be an association between the biotransformation of fluvoxamine and the activity of CYP1A2. It could not be excluded, however, that other factors may account for such a difference. Reasons include a possible association between smoking and fluvoxamine absorption, as well as between smoking and the elimination of fluvoxamine by other metabolic pathways. Further studies are, therefore, needed to clarify the role of CYP1A2 and other specific CYP in fluvoxamine metabolism and to elucidate which of the various metabolic steps could be dependent on CYP1A2. The recommended therapeutic reference range of fluvoxamine is 60–230 ng/mL [5]. Several studies have shown relations between plasma concentrations and clinical effects [5]. As a possible bias of these studies we highlight that most data involves Japanese individuals. This may influence the effect size, given, that this population presents quantitative differences, sometimes substantial, the various cytochrome P450 enzymes.

Assays for the SNRIs provide more consistent results than those involving SSRIs. Research on venlafaxine and duloxetine has similar designs, making result comparison easier and strengthening results. In both studies with venlafaxine, serum levels of both study groups showed no significant differences. Both pointed to a significant decrease in ODV levels. However, the pharmacodynamical effects of this are not completely understood, though many studies point to considerably weaker inhibition of serotonin and noradrenaline reuptake pumps when compared with venlafaxine itself [34]. For duloxetine, available data suggest a decrease in serum concentrations caused by tobacco consumption. In this drug, the available evidence is strong, based on multicenter randomized trials, involving a broad number of evaluated subjects. The effects of smoking status on duloxetine bioavailability can be attributed to the mechanism of duloxetine metabolism primarily by CYP1A2 enzyme. Smoking increases the expression of CYP1A2, which may explain the lower duloxetine bioavailability noted in smokers. The recommended therapeutic reference range of duloxetine is 30–120 ng/mL [5]. So far there is only a single retrospective analysis on plasma concentrations and clinical effects that has shown concentration-dependent improvement [5].

Data suggest a decrease in serum concentrations of trazodone caused by tobacco consumption and no influence in serum concentrations of trazodone’s active metabolite m-chloro-phenylpiperazine (mCPP). This reduction could be due to the enhancement of hydroxylation and N-oxidation of trazodone caused by PAH from cigarette smoke [29]. A concentration–response relationship for trazodone has not been established [29]; however, one study has suggested the presence of a linear relationship [35].

The two studies that evaluated the effect of smoking on mirtazapine’s pharmacokinetics show significantly lower mirtazapine and their main active metabolites (S-mirtazapine and R-N-desmethylmirtazapine) serum levels in smokers than in nonsmokers. In vitro tomography study has shown that CYP1A2 is involved in 8-hydroxylation and possibly N-oxidation of mirtazapine [30]. Additionally, uridine diphosphate glucuronosyltransferases, another enzyme involved in the metabolism of mirtazapine, are inducible by smoking [30]. The recommended therapeutic reference range of mirtazapine is 30–80 ng/mL [5]. In a study on patients with depression, responders to mirtazapine treatment presented higher plasma concentrations than non-responders [36].

In the study with bupropion serum levels, both study groups showed no significant differences [32]. This study only evaluated daily doses of 150 mg of bupropion, reporting nothing on higher doses of drug.

Most antidepressants adverse effects are dose dependent, and some arise only when serum antidepressant levels reach a certain value [5]. Given that inhibition of CYP1A2 by tobacco smoke may decrease serum levels of some drugs, smoking cessation in heavy smokers taking such medication might lead to increased serum levels. Such an increase may cause adverse effects hitherto absent.

As mentioned above, the most frequent bias found was related with selection bias, which can lead to an over/underestimation of the obtained results. Increasing the sample size and the use of control groups are recommended strategies to decrease this risk in future studies.

## Conclusions

Despite numerous limitations in most studies, available evidence indicates a reduction in the concentration of serum levels of fluvoxamine, duloxetine, trazodone and mirtazapine in smoking patients when compared to nonsmokers. These differences raise the possibility of a semi-directed choice in antidepressant treatments, adapting the dose of these drugs and being aware of possible appearances of side effects after smoking cessation.

A personalized pharmacological treatment of depression could be made possible in a nearby future, guided by increasingly common and less expensive genotyping tools. For now, treatment personalization could be based on identifying phenotypes or external variables that influence antidepressant response or side effects. Further research is needed to improve our knowledge on the influence of smoking in depression pharmacological treatment.
